# Multidimensional Pre-Competition Stress Profiles in Wheelchair Basketball: Associations with Cortisol and Psychological Indicators

**DOI:** 10.3390/life16101704

**Published:** 2026-10-11

**Authors:** Alperen Şanal, Ahmet Kurtoğlu, Sonay Serpil Daşkesen, Monira I. Aldhahi

**Affiliations:** 1 Independent Researcher, Çanakkale 17000, Türkiye; alperensanal48@gmail.com; 2 Department of Coaching Education, Faculty of Sport Science, Bandırma Onyedi Eylül University, Balıkesir 10245, Türkiye; kurtogluahmet18@gmail.com; 3 Department of Recreation, Faculty of Sport Science, Atatürk University, Erzurum 25000, Türkiye; sonay.alpdogan@atauni.edu.tr; 4 Department of Rehabilitation Sciences, College of Health and Rehabilitation Sciences, Princess Nourah bint Abdulrahman University, P.O. Box 84428, Riyadh 11671, Saudi Arabia

**Keywords:** pre-competition cortisol, stress profiles, wheelchair basketball, person-centred analysis, pre-competition anxiety, perceived stress

## Abstract

This study examined pre-competition cortisol levels and psychological anticipation in wheelchair basketball athletes using a person-centred approach. Thirty-four male athletes (mean age: 35 ± 7.75 years) competing in a regional league provided saliva samples 35–50 min before competition and completed psychological assessments with the Revised Competitive State Anxiety Inventory-2 (CSAI-2R) and the Perceived Stress Scale. K-Means cluster analysis (k = 3), validated by Ward’s hierarchical clustering (ARI = 0.947; NMI = 0.892) and Bootstrap Jaccard stability (0.544–0.603), identified three exploratory stress profiles: Low Reactor (*n* = 5; low cortisol, anxiety, and stress), High Reactor (*n* = 20; moderate cortisol, high cognitive/physical anxiety), and Physiological Stress Reactor (n = 9; high cortisol, high stress, moderate anxiety). Significant group differences were observed for log(Cortisol) (H(2) = 17.095, *p* < 0.001, ε^2^ = 0.518), Cognitive Anxiety (H(2) = 13.900, *p* < 0.01, ε^2^ = 0.421), Somatic Anxiety (H(2) = 18.840, *p* < 0.001, ε^2^ = 0.571), and Perceived Stress (H(2) = 21.831, *p* < 0.001, ε^2^ = 0.662), while self-confidence did not differ. Exploratory logistic regression indicated that functional classification, years of sport participation, match location, and match result did not significantly predict the Physiological Stress Reactor membership. These findings suggest that pre-competition stress-related characteristics may manifest in different combinations of physiological and psychological indicators. The identified profiles should be considered exploratory and require validation in larger and independent samples.

## 1. Introduction

The psychological and physiological responses of athletes—particularly the effects of stress and anxiety on athletic performance—have long been a key area of research in sports science. In competitive sporting environments, the stress factors faced by athletes consist of two main components: physical and psychological. Factors such as the competition venue, pressure from spectators, the expectations of the coaching staff and team-mates, and the intensity of the competition are among the key factors determining the levels of stress and anxiety perceived by athletes [1]. It is known that such stress factors also influence an individual’s physiological responses and, in particular, intensify the stress response by increasing cortisol release [2,3]. Cortisol release is one of the fundamental functions of the hypothalamic–pituitary–adrenal (HPA) axis. Communication between the hypothalamus, the pituitary gland and the adrenal glands forms a hormonal cascade that mobilises the body in response to stress [4]. In the functioning of the HPA axis, when the hypothalamus detects stress, it secretes CRH; CRH stimulates the pituitary gland, triggering the production of ACTH; ACTH, in turn, activates the adrenal glands, initiating the secretion of cortisol [5]. Cortisol released during the pre-competition period has been investigated as a physiological indicator associated with stress and anticipatory responses in athletes [6,7]. However, studies conducted in recent years have shown that the cortisol–anxiety relationship is not linear or straightforward. It has been demonstrated that there are significant differences between individuals, with some athletes reporting low levels of anxiety despite high cortisol levels [8]. The investigation of stress responses in athletes with disabilities is an area that has been less extensively studied compared to able-bodied athletes. Factors such as the nature of the disability, the athlete’s lifestyle and the competitive environment may influence the stress experience and physiological responses in athletes with disabilities [9]. In particular, the functional classification system categorizes athletes according to their functional capacity in wheelchair basketball, and it is not known whether functional classification is associated with pre-competition stress profiles.

Traditional variable-centred analytical approaches examine the general and average trends within the sample. This approach overlooks the individual differences among athletes. However, stress responses exhibit a heterogeneous structure at the individual level, and person-centred approaches are required to reveal this heterogeneity [10,11]. Cluster analysis is one of the most widely used statistical methods within the person-centred approach [12]. It brings individuals’ natural differences to light. Thanks to the clustering method, sub-groups with similar characteristics can be identified beyond the scope of variable-centred analytical approaches [13]. This enables the detailed examination of each group’s unique stress responses or psychological profile.

In recent years, the concept of distinct stress response profiles has received increasing attention in sport psychology and psychoneuroendocrinology. According to this conceptual framework, individuals may exhibit different combinations of physiological and psychological characteristics in response to stress [14,15]. Four broad profiles have been described in the literature: High Reactors (high physiological and high psychological), Low Reactors (low physiological and low psychological), Psychological Reactors (low physiological and high psychological) and Physiological Reactors (high physiological and low psychological) [16,17]. However, studies have also identified profiles that differ from these four categories [18,19,20]. The presence and distribution of these types among athletes with disabilities have not yet been tested. The Physiological Reactor profile is of particular interest because it represents a pattern in which physiological and psychological indicators may not necessarily show the same level of elevation [21]. On the other hand, for people with disabilities, this situation may stem from adopting a personality different from their own due to social and societal pressures [22]. The literature suggests that people with disabilities may adopt stronger or more assertive personalities in response to social pressures, as a coping mechanism, or to demonstrate their resilience and independence [23]. For this reason, determining the physiological and psychological profile is important both theoretically, in enriching stress response models, and practically, in terms of athlete health and performance management.

The aim of the present study is to examine multidimensional pre-competition stress profiles in athletes with disabilities using a person-centred approach, with particular attention to pre-competition cortisol and psychological indicators. The original contributions of the study are as follows: (a) the identification of stress reactivity types in athletes with disabilities using cluster analysis for the first time; (b) testing the existence of the Physiological Reactor profile; (c) examining the relationship between functional classification and sport-specific characteristics and these profiles; and (d) selecting predictors of cortisol levels using Lasso regression. To this end, we hypothesized that distinct clusters of athletes would emerge based on their pre-competition cortisol and psychological indicators, reflecting different multidimensional stress profiles.

## 2. Materials and Methods

### 2.1. Study Design, Setting and Participants

The study employed a multi-subject experimental design, one of the quantitative research methods. Thirty-four athletes from four wheelchair basketball teams competing in a regional league took part in the study. The sample comprised thirty-four male athletes aged between 21 and 47, all of whom were actively participating in a regional wheelchair basketball league. Female athletes were excluded for two main reasons: (1) they represent only approximately 2 per cent of the league’s total athlete population, which limits their statistical representativeness; and (2) as the existing literature reports gender differences in stress responses and cortisol responses, combining male and female athletes could confound the results. In particular, some studies suggest that female athletes exhibit higher stress responses compared to males, and that cortisol levels in women may be inversely proportional to body fat percentage [24]. Whilst this selection limits the gender inclusivity of the findings, it was necessary to ensure hormonal homogeneity within the sample.

All participants had at least three years’ experience in wheelchair basketball (Table 1). The inclusion criteria required participants to be active, competitive athletes playing in official matches; athletes who remained on the substitutes’ bench and did not take part in matches were excluded. To control for external influences on psychological variables, participants were prohibited from using supplements (e.g., multivitamins, protein, BCAAs, L-carnitine, antioxidants) throughout the study period. This restriction is based on previous research indicating that such supplements may influence psychological outcomes such as anxiety and stress [25]. Furthermore, participants were required to maintain their usual dietary habits and to refrain from using anti-inflammatory medication or participating whilst suffering from any illness.

All participants provided written informed consent after being informed about the study procedures. The study was conducted in accordance with the ethical principles set out in the Declaration of Helsinki and received approval from the Ethics Committee of Atatürk University, Erzurum (Approval No: E-70400699-000-2500027585, 2025/1).

### 2.2. Assessments

For the study, psychological and physiological assessments of the anxiety states of wheelchair basketball players were carried out. The Competitive State Anxiety Inventory-2 (CSAI-2) and the Anxiety/Stress Scale were used for psychological assessments. Cortisol levels obtained from saliva samples were used for physiological assessments. The Competitive State Anxiety Inventory-2 (CSAI-2) and the Anxiety/Stress Scale were administered 1 h before the competition. Saliva samples for physiological evaluation were collected before the athletes began their warm-up (approximately 30–45 min beforehand). Data were collected from four different teams (two away teams and two home teams) during the second half of the wheelchair basketball league.

Anthropometric Measurements: Body mass was assessed using an electronic scale (seca GmbH & Co. KG, Hamburg, Germany) whilst the athletes were seated in their wheelchairs. To obtain an accurate body weight, the standard reference weight of the wheelchair (approximately 12.5 kg) based on the typical wheelchair weight used in Turkish wheelchair basketball was subtracted from the total measured weight [26]. Seated height was measured using a precision tape measure (accuracy of 0.1 cm) whilst the athletes were in their sports wheelchairs [27].

Competitive State Anxiety Inventory-2 (CSAI-2): The CSAI-2 is a simple and valid tool commonly used to assess pre-competition anxiety in athletes [28,29]. Originally developed in 1990 [30], the scale has since been revised by various researchers to improve its psychometric properties. It evaluates three key dimensions: cognitive anxiety, somatic anxiety and self-confidence. The scale consists of 27 items in total. There are three subscales in the scale. These are cognitive anxiety (9 items), somatic anxiety (9 items) and self-confidence (9 items). It employs a 4-point Likert-type response scale, ranging from 1 (not at all) to 4 (very much). Higher scores on the cognitive and somatic anxiety subscales indicate higher levels of anxiety, whilst higher scores on the self-confidence subscale reflect greater confidence prior to competition. The scale was adapted into Turkish in 2021 [31]. The Turkish revised version, referred to as the Revised Competition State Anxiety Inventory-2 (CSAI-2R) in the English version of the validation study and as the Revize Edilmiş Yarışma Durumluk Kaygı Envanteri-2 (YDKE-2R) in Turkish, was reduced to 14 items. The present study used this Turkish revised version rather than the original 27-item CSAI-2. The revised version comprises three subscales: cognitive anxiety (4 items), somatic anxiety (5 items) and self-confidence (5 items). The scale also contains reverse-scored items. These are the self-confidence items. Following the reliability analysis of the scale, the Cronbach’s alpha (α) coefficient was calculated as 0.71 for the cognitive anxiety subscale, 0.78 for the physical anxiety subscale, and 0.80 for the self-confidence subscale [31]. As a result of the internal consistency analysis conducted for this study, the Cronbach’s alpha (α) coefficient of the scale for this study was calculated as 0.81 for the cognitive anxiety subscale, 0.83 for the physical anxiety subscale, and 0.89 for the self-confidence subscale.

Perceived Anxiety/Stress Scale: A single-item perceived anxiety/stress rating was developed using a 10-point response format conceptually based on the Borg perceived exertion scale, which provides a simple numerical framework for rating subjective experience. The rating was used as an exploratory measure of athletes’ immediate subjective stress-related state rather than as a validated diagnostic or psychometric measure of anxiety or stress. Athletes were asked to provide a global rating of their current subjective stress-related state immediately before competition using the question: “How anxious/stressed do you feel right now? Please rate from 1 to 10, where 1 indicates the lowest and 10 the highest level.” The terms anxiety and stress were used together to capture a broad subjective pre-competition stress-related state rather than to imply that they represent the same psychological construct. Responses were recorded on a 10-point scale.

Salivary Cortisol Sampling: Saliva samples were collected approximately 35–50 min before the scheduled start of the match, immediately before the players began their warm-up. This timing was selected to minimize the potential influence of acute physical exertion associated with the warm-up on cortisol concentrations. All matches were scheduled to start at 14:00, and saliva samples were therefore collected between 13:10 and 13:25. Before saliva collection, participants were asked whether they had used any stimulant or sedative medication or supplements immediately before the competition, and no participant reported such use. Saliva samples were collected in 10 mL tubes (Sarstedt AG & Co. KG, Nümbrecht, Germany) provided by the hospital, and the athletes’ identification numbers were written on the tubes before collection. Immediately after collection, the tubes were placed in a dedicated saliva transport container and delivered to the hospital. As all matches were held on Sunday, the saliva samples were stored at −20 °C overnight in a laboratory freezer (Thermo Fisher Scientific, Waltham, MA, USA) and processed on the following day using the same laboratory procedure for all samples. The samples were thawed, centrifuged at 1500× *g* for 15 min using a laboratory centrifuge (Eppendorf SE, Hamburg, Germany), and the supernatants were collected and stored at −80 °C until analysis [32]. Samples were tested for cortisol concentration using a commercially available enzyme-linked immunosorbent assay (ELISA) kit (Salimetrics LLC, Carlsbad, CA, USA) in accordance with established procedures [33,34]. All samples were tested on a single assay plate.

### 2.3. Sample Size

Given the exploratory nature of this profiling study and limited access to wheelchair basketball athletes, the sample size was determined by feasibility and voluntary participation rather than a priori power calculations. Participants were recruited from four Regional League teams. Unsupervised cluster analysis does not require equal pre-specified group sizes; therefore, the cluster sizes emerged from the observed data. The final sample comprised 34 athletes and yielded three clusters of unequal sizes (n = 5, 9, and 20) [35,36].

### 2.4. Statistical Analysis

Descriptive statistics were used to analyse the demographic and sporting characteristics of the wheelchair basketball players participating in the study. There were no missing values in the dataset (logCortisol). Normality was assessed using the Shapiro–Wilk test. As the data exhibited a right-skewed distribution, a natural logarithmic transformation was applied. Findings in the literature indicate that salivary cortisol levels exhibit a right-skewed distribution, a phenomenon explained by differences in secretion rates and variability in cortisol-binding globulin (CBG) concentrations [37]. Homogeneity of variance was assessed using the Levene test.

A cluster analysis using K-Means clustering was performed. Five variables were included: log Cortisol Pre, Cognitive Anxiety Pre, Bodily Anxiety Pre, Self-Confidence Pre, and Perceived Stress Pre. The Elbow method and average Silhouette width were used together to determine the optimal number of clusters. The K-Means algorithm was run with k = 3, nstart = 25, and seed = 123. Cluster sizes were determined by the observed data and were not pre-specified.

Ward’s Hierarchical Clustering analysis was carried out to validate the K-Means clustering analysis. Cluster correspondence was assessed using the Adjusted Rand Index (ARI) and Normalized Mutual Information (NMI). A bootstrap-stabilised analysis was also carried out to assess the stability of the clustering solution; the Jaccard stability index was calculated using B = 100 bootstrap samples. According to the Jaccard index, the three clusters were found to exhibit moderate stability (Jaccard: 0.544–0.603).

In the between-group comparison, a Kruskal–Wallis non-parametric test was used due to the small sample size in one group (n = 5). Epsilon-squared (ε^2^) was used as the effect size for the Kruskal–Wallis test, and Cliff’s delta was used for post hoc comparisons. An exploratory logistic regression analysis was conducted to examine the associations of Classification High (≥3.5 points = 1, <3.5 points = 0), Sport Year, Home/Away (Away = 1), and Win/Lose (Lose = 1) with Physiological Stress Reactor membership. The dependent variable was coded as Physiological Stress Reactor (1) versus other clusters (0). Independent variables: Classification High (≥3.5 points = 1, <3.5 points = 0), Sport Year, Home/Away (Away = 1) and Win/Lose (Lose = 1). Ridge and Lasso regressions were applied to select the factors associated with cortisol levels and optimal λ values were determined using 5-fold cross-validation.

All analyses were carried out using R (version 4.5.x; R Foundation for Statistical Computing, Vienna, Austria). The level of statistical significance was set at α = 0.05.

## 3. Results

Table 1 presents the sporting, physiological and psychological characteristics of the wheelchair basketball players participating in the study. The mean weight of all participants was 72.14 ± 10.14, the mean height was 142.14 ± 8.73, the mean age was 35.0 ± 7.74, the mean sport age was 9.79 ± 3.81, average classification score was 2.73 ± 1.03, average pre-test cognitive anxiety score was 11.11 ± 1.78, average pre-test bodily anxiety score was 8.79 ± 1.51, average pre-test self-confidence score was 16.61 ± 1.41, the mean pre-test perceived stress score was 3.64 ± 1.61 and the mean pre-test cortisol level was 10.02 ± 2.09.

**Figure 1:**
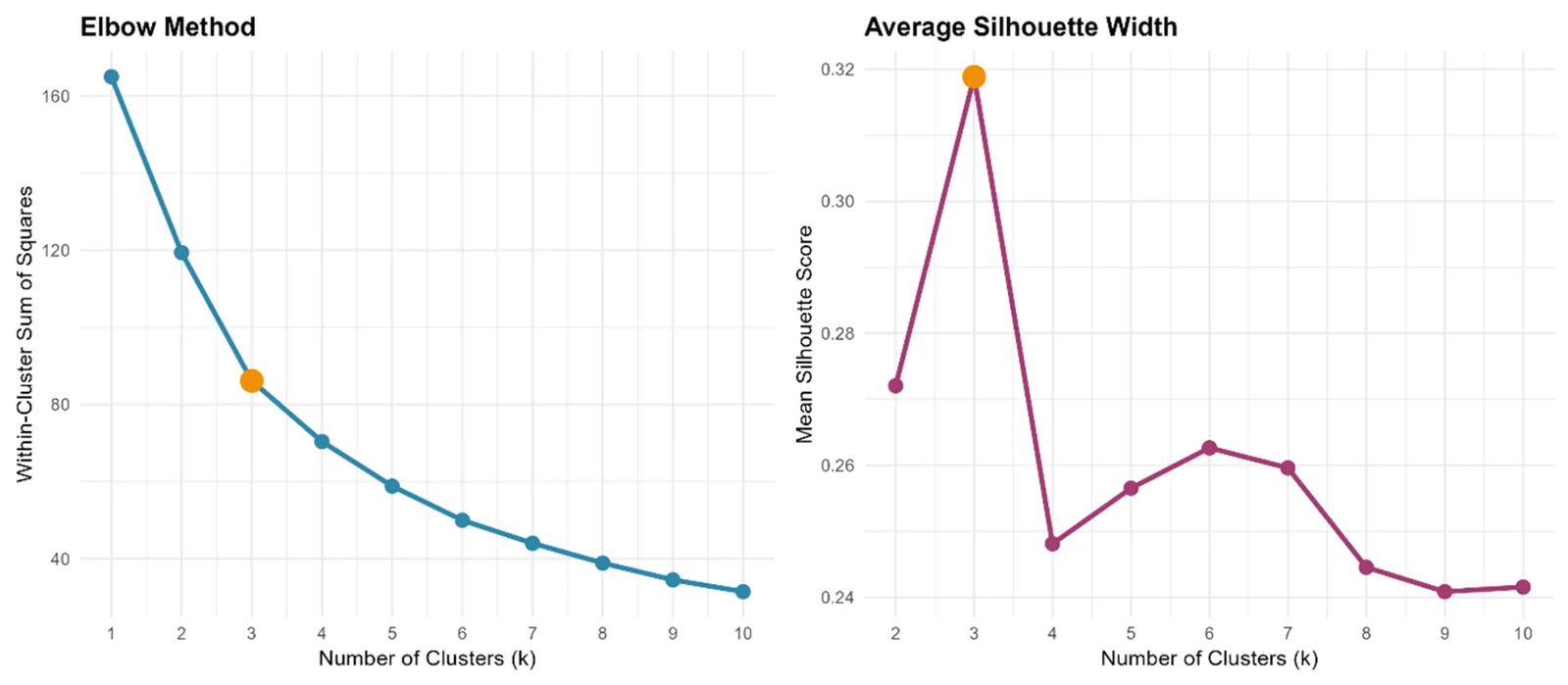
Determination of the optimal number of clusters using the Elbow Method and Average Silhouette Width Method. The left panel illustrates the within-cluster sum of squares (WCSS) across different numbers of clusters (k), with the orange circle highlighting the selected elbow point at k = 3. The right panel presents the average silhouette width for different cluster solutions, with the orange circle indicating the highest silhouette score (approximately 0.319) at k = 3. Both methods support the selection of a three-cluster solution for subsequent analyses.

The Elbow plot showed a marked slowdown in the decline of the total within-cluster sum of squares (WCSS) after k = 3. The average Silhouette score was highest for k = 3 (0.32), compared with 0.27 for k = 2 and 0.24–0.26 for the other solutions. Therefore, a three-cluster solution was selected (Figure 1).

**Figure 2:**
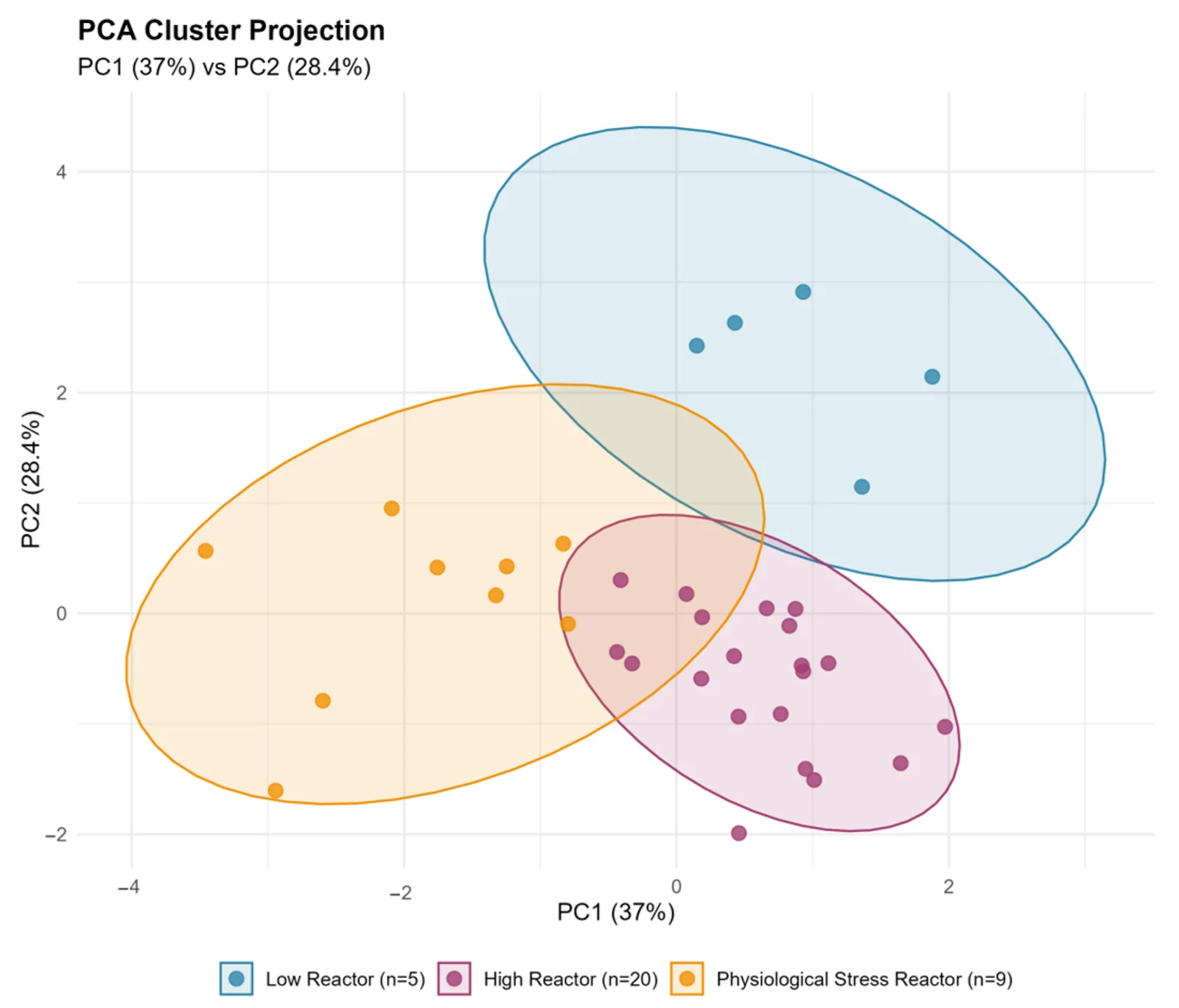
PCA Cluster Projection.

When the PCA projections for the three stress reactor groups are examined in terms of physiological and psychological variables in Figure 2, PC1 (37 per cent) and PC2 (28.4 per cent) account for 65.4 per cent of the total variance. Distinct differences are observed between the groups. Accordingly, the Low Reactor group (n = 5) is located on the positive axis of both PC1 and PC2; the High Reactor group (n = 20) is located on both the positive and negative axes of PC1 and PC2; and the Physiological Stress Reactor group (n = 9) is located on the negative axis of PC1 and on both the positive and negative axes of PC2 (Figure 2).

**Figure 3:**
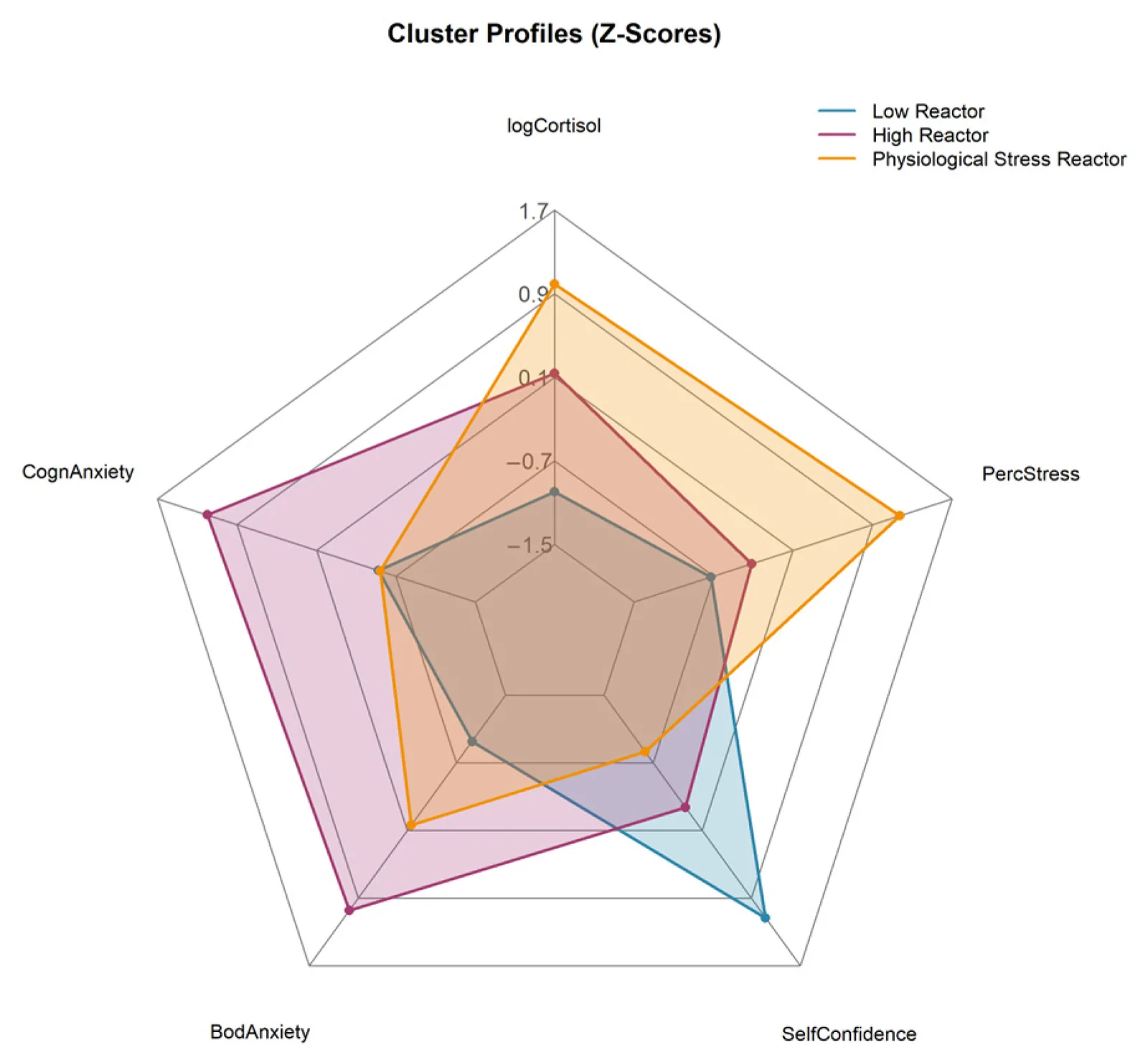
Cluster Profiles (Z-Scores).

Figure 3 shows the standardised (Z-scores) physiological and psychological profiles of the three stress reactor groups. The group with the highest logCortisol Z-score is the Physiological Stress Reactor (1.0), whilst the group with the lowest Z-score is the Low Reactor (−1.0). The group with the highest PercStress score is the Physiological Stress Reactor (1.1), whilst the group with the lowest Z-score is the Low Reactor (−0.7). The group with the highest Self-Confidence Z-score is the ‘Low Reactor’ (1.1), whilst the group with the lowest Z-score is the ‘Physiological Stress Reactor’ (−0.6). The group with the highest BodAnxiety Z-score is the ‘High Reactor’ (1.0), whilst the group with the lowest Z-score is the ‘Low Reactor’ (−0.5). It can be seen that the group with the highest CogAnxiety Z-score is the High Reactor (1.2), whilst the groups with the lowest Z-scores are the Low Reactor (−0.5) and the Physiological Stress Reactor (−0.5) (Figure 3).

Table 2 presents the demographic, sporting, physiological and psychological characteristics of the three stress reactor groups. In terms of height, the physiological stress reactor group is the tallest compared to the other groups. In terms of weight, the low reactor group is the heaviest compared to the other groups. In terms of age, the Low Reactor group (43.00 ± 4.90 years) is older than the High Reactor (34.85 ± 6.61) and Physiological Stress Reactor (30.89 ± 8.54) groups. There is no noticeable difference between the groups in terms of years of sport participation and classification score. In physiological measurements, higher pre-competition cortisol levels were 7.40 ± 0.89 nmol/L for the Low Reactor group, 9.80 ± 1.61 nmol/L for the High Reactor group and 12.00 ± 1.66 nmol/L for the Physiological Stress Reactor group. Similarly, perceived stress scores also increase in the same order (2.20 ± 0.45, 3.00 ± 0.79 and 5.89 ± 1.05 points, respectively). Whilst cognitive anxiety levels were highest in the High Reactor group (12.05 ± 1.43), physical anxiety levels also peaked in this group (9.65 ± 1.14). Self-confidence scores showed a relatively similar distribution across the groups. The distribution of home match percentages differed between the groups. In the Physiological Stress Reactor group, the proportion of home matches (55.6 per cent) is higher than in the other groups; in the Low Reactor group, this proportion is at its lowest (20 per cent). In contrast, the proportion of away matches (80 per cent) in the Low Reactor group is higher than in the other groups. The distribution of match results appears to be relatively homogeneous across the groups.

The Shapiro–Wilk test indicated deviations from normality for log(Cortisol) in the Low Reactor group (*p* = 0.042), Bodily Anxiety in the Physiological Stress Reactor group (*p* = 0.012), Self-Confidence in the High Reactor group (*p* = 0.044), and Perceived Stress in the Low Reactor (*p* < 0.001) and High Reactor (*p* = 0.003) groups.

The analysis of the physical and psychological measures of the three stress reactor groups presented in Table 3 revealed significant differences in log(cortisol), Cognitive Anxiety, Bodily Anxiety and Perceived Stress (*p* < 0.05). Based on these findings, the Physiological Stress Reactor group had the highest log(cortisol) score, whilst the Low Reactor group had the lowest. In terms of Cognitive Anxiety, the High Reactor group had the highest mean, whilst the Low Reactor and Physiological Stress Reactor groups had the lowest means. For Bodily Anxiety, the High Reactor group had the highest mean, whilst the Low Reactor group had the lowest. For Perceived Stress, the Physiological Stress Reactor group had the highest mean, whilst the Low Reactor group had the lowest.

No significant differences were found between the groups in terms of Self-Confidence (*p* > 0.05).

**Figure 4:**
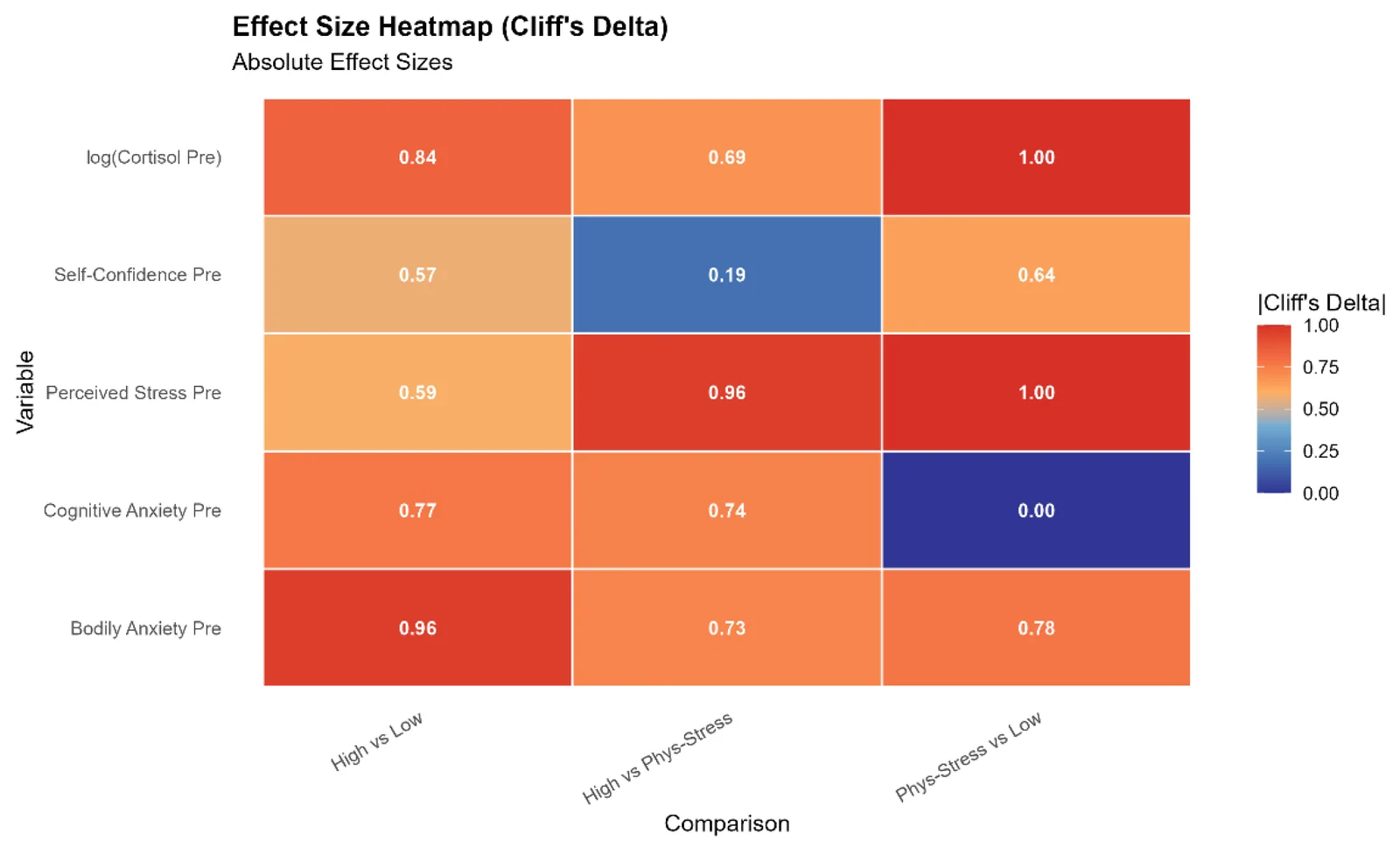
Cliff’s Delta absolute effect size values for comparisons between the three stress reactor groups (heatmap).

In the distributions of effect sizes (Cliff’s Delta) across groups, the difference between Physiological Stress Reactors and Low Reactors on the log(Cortisol) variable exhibits the highest effect size (|d| = 1.00). In Bodily Anxiety, the comparison between the High Reactor and Low Reactor groups (|d| = 0.96) and, in Perceived Stress, the comparison between the High Reactor and Physiological Stress Reactor groups (|d|= 0.96) exhibit the highest effect sizes. In Cognitive Anxiety, the effect size between the ‘Physiological Stress Reactor’ and ‘Low Reactor’ groups is close to zero (|d| = 0.00). Furthermore, no significant differences were detected in the post hoc analyses (*p* > 0.05). For Self-Confidence, the effect size between the High Reactor and the Physiological Stress Reactor groups is low (|d| = 0.19). No significant differences were found between the three groups in the post hoc analyses (Kruskal–Wallis H(2) = 5.266, *p* = 0.072) (Figure 4).

**Figure 5:**
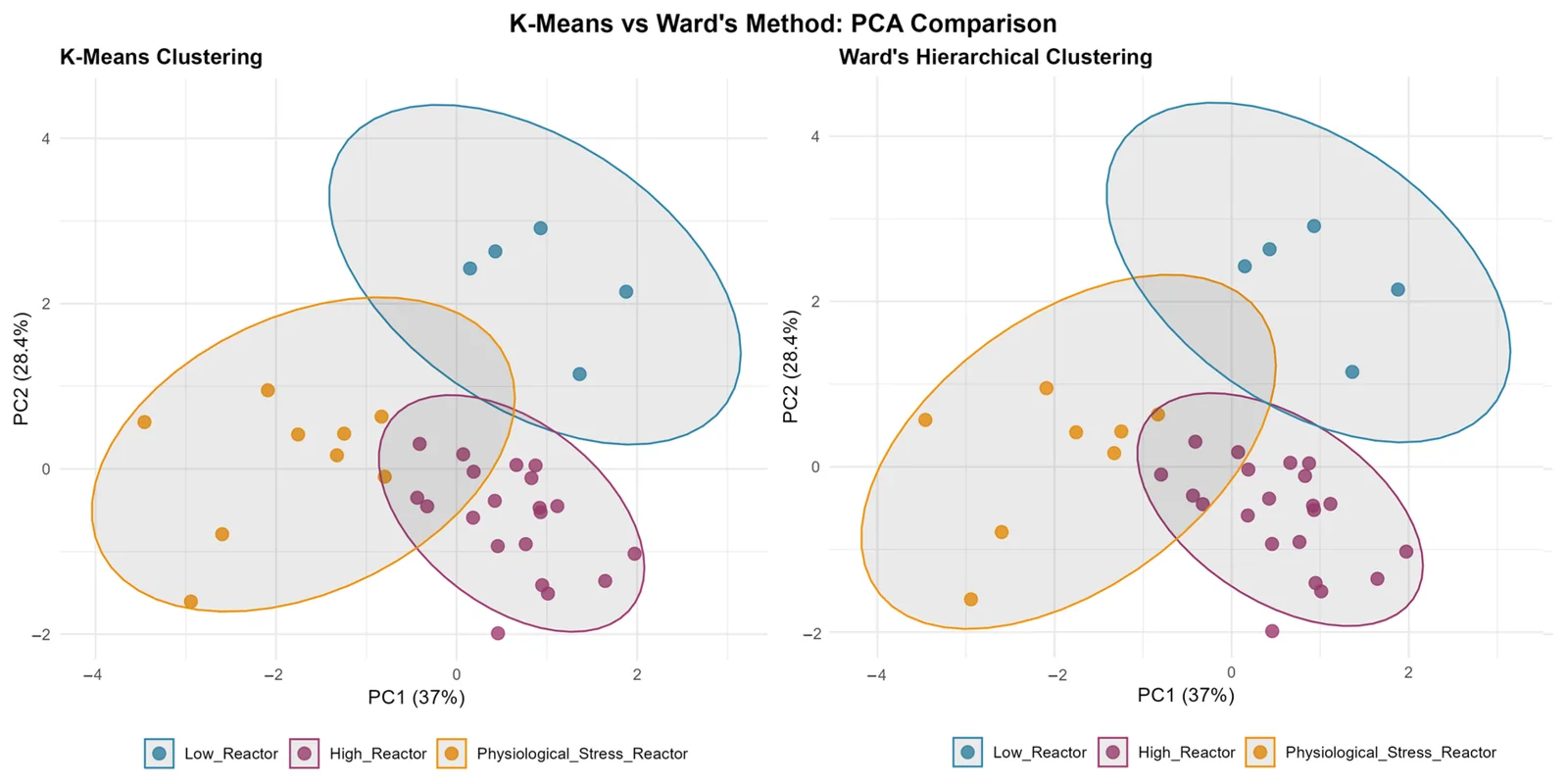
Comparison of K-Means (**left**) and Ward’s hierarchical clustering (**right**) results via PCA projection.

Ward’s hierarchical clustering confirmed the K-Means solution with 97.1 per cent agreement between the two methods (ARI = 0.947; NMI = 0.892). According to Ward’s method, five participants were identified as ‘Low Reactor’, 21 as ‘High Reactor’ and eight as ‘Physiological Stress Reactor’. The Adjusted Rand Index (ARI) was 0.947 and the Normalised Mutual Information (NMI) was 0.892, indicating almost perfect agreement beyond chance (Figure 5).

**Figure 6:**
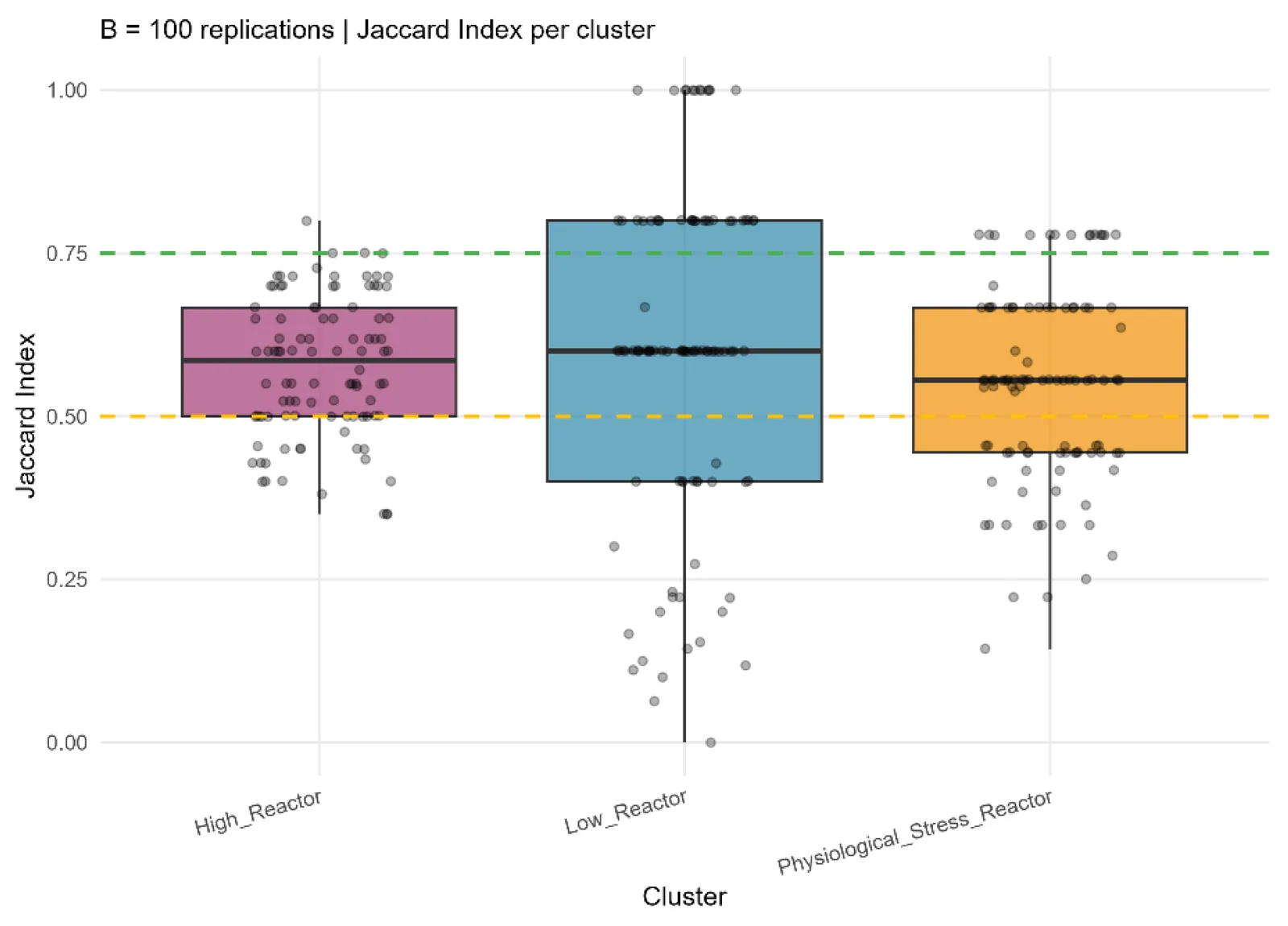
Bootstrap Stability Distribution.

The bootstrap Jaccard stability analysis showed that all three clusters exhibited limited-to-moderate stability (Jaccard: 0.544–0.603). The highest stability was observed in the Low Reactor cluster (0.603), whilst the lowest stability was observed in the Physiological Stress Reactor cluster (0.544) (Figure 6).

An exploratory logistic regression analysis was conducted to examine the associations of classification experience, sport year, match location, and match result with Physiological Stress Reactor membership (Table 4). None of the predictors was statistically significant (all *p* > 0.05). Given the small number of Physiological Stress Reactor cases (n = 9) relative to the number of predictors, these findings should be interpreted cautiously.

The Ridge regression produced small non-zero coefficients across all predictors (range: −0.0017 to 0.0035). In the penalized regression analysis, LASSO selected Perceived Stress as the only non-zero predictor of cortisol levels at the minimum cross-validated penalty (λmin = 0.0361; β = 0.0467). The coefficients for Cognitive Anxiety, Bodily Anxiety, Self-Confidence, and Classification Point were shrunk to zero. The minimum cross-validation error was 0.0369, with a deviance ratio of 0.332.

## 4. Discussion

The results of this study indicate that stress reactivity in wheelchair basketball players cannot be explained by a single biological or psychological axis, but rather represents a heterogeneous psychophysiological state. The study identified three stress reactor groups, revealing differences between these groups in terms of log-cortisol, cognitive anxiety, somatic anxiety and perceived stress. A key finding of this study is that, despite athletes competing within the same league context, their psychological and physiological stress indicators do not move in the same direction. In other words, assessments based on a single stress marker may mask important individual patterns of actual stress reactivity. For this reason, rather than classifying the stress response unidimensionally as ‘high’ or ‘low’ based on a single indicator, analysing it through multidimensional profiles formed by different biological and psychological components leads to more explanatory results [38].

The research findings showed that the three groups differed in terms of the predominant component of their overall stress response. In the low-reactor group, cortisol, cognitive and physical anxiety, and perceived stress were at low levels; in the high-reactor group, cognitive and physical anxiety were at the highest levels; and in the Physiological Stress Reactor group, cortisol and perceived stress were at the highest levels (Table 3). The tripartite structure derived from these findings points to a distribution of profiles in which different components of stress can become dominant in different athletes, rather than a simple ‘from low to high’ ranking of stress [39,40]. This pattern suggests that, whilst the biological, cognitive and subjective components of stress may be interrelated, they are not direct equivalents of one another. This contrasting pattern suggests that psychological stress should not be used as a simple substitute for physiological stress, just as a single biological indicator cannot fully reflect an individual’s subjective experience of stress [41,42].

In the literature, the stress reactor typology is commonly defined as a four-dimensional approach comprising the categories ‘low’, ‘high’, ‘physiological’ and ‘psychological’ [16,17]. In wheelchair basketball players, however, the data reveal a three-profile structure derived from common patterns of variation within the data itself, rather than a direct replication of a predefined typology. It is therefore more appropriate to regard the groups defined here not as ‘pre-validated biological classes’, but as empirical stress profiles emerging from the current set of measurements and sample. However, the fact that the classic psychological reactor profile did not emerge as a distinct cluster in this study should not be interpreted as meaning that this phenotype is absent from the population. This may be due to the sample size and the nature of the sample group (Second Division teams). Nevertheless, there are studies in the literature that support our research findings, in which different typologies from these four have been identified [18,19,20].

The research findings revealed significant differences in cortisol levels, as assessed biologically, between the groups. The Physiological Stress Reactor group had the highest pre-competition cortisol levels, the High Reactor group had moderate levels, and the Low Reactor group had the lowest levels. The significant difference between the groups and the high effect size indicate that the divergence between the groups is not merely statistical but represents a distinct effect. However, it would be inappropriate to interpret the group with the highest cortisol levels as having HPA axis hyperactivity. This is because the study was measured at a single time point (pre-match). This provides an indicator of the endocrine state of the that specific moment only and does not reflect the HPA axis’s reactivity, diurnal rhythm or dynamic response to stressors [43,44]. Cortisol levels can also be influenced by factors such as time of day, circadian rhythm, physical exertion, sleep and diet [44]. Consequently, the Physiological Stress Reactor group showed higher pre-competition cortisol levels than the other groups, consistent with a more pronounced physiological stress component [38]. However, further research is needed to determine whether this pattern reflects sustained or altered HPA-axis activity [40]. Furthermore, the Physiological Stress Reactor group reports the highest levels of perceived stress compared to the other groups. Thus, both the biological stress marker and subjective perceived stress were elevated in this profile. However, this does not definitively indicate that high cortisol leads to high perceived stress, or that high perceived stress causes an increase in cortisol [45,46]. In this context, it can be interpreted that the two indicators may reflect different levels of a shared stress burden in the same individuals. The fact that cognitive anxiety does not differ between the Physiological Stress Reactor group and the Low Reactor group indicates that high cortisol and perceived stress do not necessarily overlap with cognitive anxiety. The distinguishing feature of this profile is not ‘high anxiety’, but rather the physiological–subjective pattern in which higher pre-competition cortisol is observed alongside high perceived stress [38]. This highlights the limitations of single-indicator stress assessments. This is because a single-indicator stress assessment may fail to capture either the physiological burden or psychological anxiety [45]. Consequently, the combined assessment of physiological and psychological indicators provides a more comprehensive understanding of pre-performance stress at the individual level [40].

The High Reactor group demonstrates the clearest distinction between physiological and psychological stress indicators. Although cognitive and physical anxiety are at their highest levels in this group, cortisol and perceived stress levels lag behind those of the Physiological Stress Reactor group. Consequently, the High Reactor profile can be interpreted as a pattern in which psychological anxiety predominates but is not accompanied by a strong cortisol response [38]. The fact that cognitive anxiety reaches its highest level in this group may be related to the perception of threat, expectations of failure and mental preoccupation processes in a performance context [30]. However, this does not imply that individuals in the High Reactor group perform poorly. The fact that physical anxiety is also at its highest level indicates that cognitive and somatic anxiety can co-occur in the same individuals. However, it should be borne in mind that this is a subjective measure and does not directly represent physiological activation. Therefore, physical anxiety should be assessed as part of the psychological stress pattern based on the somatic symptoms perceived by the individual [47].

The Low Reactor group is defined as having the lowest reactivity profile, characterised by low cortisol levels, low cognitive and physical anxiety, and low perceived stress levels. Although the average self-confidence score is highest in this group, the difference is not statistically significant. Consequently, self-confidence should be regarded as a concomitant characteristic rather than a validated predictor. The fact that self-confidence does not show significant differences across stress reactors suggests that there is no direct relationship between stress reactivity and self-assessment of performance. This may be explained by self-confidence being a more stable psychological construct or by the measurement having limited sensitivity to short-term differences in stress; however, the present study is unable to distinguish between these mechanisms [30,48]. This is because self-confidence was assessed as a subscale of the CSAI-2R. Consequently, the assessment of self-confidence independently of other stress markers (cortisol, heart rate, etc.) is limited. For this reason, the self-confidence parameter needs to be re-examined in future studies using independent measurement tools [49].

One of the study’s strengths is that the stress profiles do not rely on a single algorithm, and a 97.1 per cent agreement (ARI = 0.947; NMI = 0.892) was found between K-means and Ward clustering. This supports internal robustness [50,51]. However, this should not be confused with external validity. The fact that different algorithms yield similar results in the same sample does not guarantee that the same profiles will emerge in other groups of athletes. The fact that the bootstrap Jaccard values fall within the range of 0.544–0.603 indicates that the clusters are not entirely invariant under resampling and that the Physiological Stress Reactor group, in particular, may be more heterogeneous [50,52]. Consequently, high ARI/NMI values combined with moderate Jaccard stability are not contradictory but reflect different aspects. These findings support interpreting the tripartite stress reactor solution is an exploratory yet methodologically sound model, aimed at identifying meaningful stress patterns within the current sample rather than defining universal biological phenotypes. External validity, however, must be tested using independent samples [53,54]. The PCA results also support this multivariate structure, explaining 65.4 per cent of the variance and providing additional structural support for the observed profile differentiation. However, PCA is not an independent validation but rather a visual-structural support for the current solution [55].

The fact that classification point, sporting year, match location and match result do not significantly predict being a Physiological Stress Reactor indicates that stress profiles cannot be explained solely by sporting experience or the match context [56]. Similarly, the lack of significance for the classification point and year of sport does not imply that these variables are unrelated to stress reactivity. It can be interpreted as meaning that, within the current sample size and model structure, they do not provide independent explanatory evidence [57]. When the study’s findings are evaluated collectively, they emphasise that understanding stress reactivity requires not merely determining the extent of stress, but also identifying in which components and combinations it manifests. The ‘Low Reactor’ is characterised by low physiological and psychological reactivity, the ‘High Reactor’ by predominant psychological anxiety, and the ‘Physiological Stress Reactor’ by high cortisol levels and perceived stress. This differentiation suggests that athletes in the same context may organise their stress responses in different ways. For this reason, assessments should be based on multiple indicators rather than single indicators [38].

The study’s strengths include the assessment of physiological and psychological indicators within the same framework, the comparison of different clustering methods, the examination of the multivariate structure using PCA, and the application of bootstrap stability analysis. This approach enabled a holistic examination of stress patterns rather than a single-variable analysis [50]. However, the sample size was limited by accessibility and voluntary participation, and no a priori power analysis was performed. The Low Reactor cluster included only five athletes, which limits cluster-specific inferences and subgroup stability. Cluster sizes were data-driven and not imposed a priori; therefore, the profiles should be considered exploratory and interpreted cautiously. Strong effect sizes should be confirmed in independent samples [58]. The exploratory logistic regression analysis included only nine Physiological Stress Reactor cases and four predictors; therefore, the estimates and confidence intervals may be unstable and should be interpreted cautiously. As a cross-sectional design and a single-time-point cortisol measurement cannot capture zortisol dynamics, cortisol was assessed only once, immediately before the warm-up; therefore, cortisol reactivity and changes from baseline to post-competition could not be assessed. The findings should therefore be interpreted as pre-competition or anticipatory cortisol levels. Although all matches were scheduled at 14:00, diurnal variation cannot be completely excluded. Other potential factors that may influence cortisol concentrations, including endocrine conditions, regular medication use, sleep, and dietary intake, smoking and alcohol use were not systematically assessed. Although participants reported no pre-competition use of stimulant or sedative medication or supplements, these additional factors may have contributed to individual variation in pre-competition cortisol concentrations. Given the limited and highly specific population of wheelchair basketball athletes available for recruitment, applying extensive exclusion criteria based on such factors would have further reduced the accessible sample. Therefore, these factors were not used as exclusion criteria but should be considered potential sources of individual variation in pre-competition cortisol concentrations. Future studies with larger and independently recruited samples should systematically assess these factors and, where feasible, account for them in the study design and statistical analyses Future studies should also time measurements appropriately and assess additional endocrine/autonomic markers [45]. Furthermore, objective game-performance variables were not collected; therefore, the practical or predictive relevance of the stress profiles for competitive performance could not be examined. Future studies should relate these profiles to objective performance indicators [49]. Although the current approach is robust in terms of internal validity, it is limited in terms of external validity. Reproducing the study across different league levels and with different samples is an important research priority, particularly regarding the generalisability of the Physiological Stress Reactor profile [54]. The medical aetiology of the athletes’ disabilities was not systematically recorded; therefore, its potential influence on stress profiles could not be examined. Future studies should include detailed disability-related characteristics alongside IWBF functional classification. Furthermore, all participants in the study were male athletes. Therefore, sex differences could not be examined. However, this comparison is difficult in Turkish leagues because women account for only 2 per cent of the total number of athletes in the league.

## 5. Conclusions

In conclusion, this study suggests that pre-competition stress profiles in wheelchair basketball players is not a homogeneous construct, and that physiological, psychological and subjective indicators can manifest in different combinations across individuals. The ‘Low Reactor’ is characterised by low overall reactivity, the ‘High Reactor’ by predominant cognitive and physical anxiety, and the ‘Physiological Stress Reactor’ by high cortisol levels and perceived stress [59]. A key contribution of the present study is the observation that stress-related responses may not be adequately characterized along a single axis and that single-indicator approaches may mask individual differences. Therefore, multimodal assessment may contribute to a more accurate understanding of pre-performance psychophysiological load [38]. Rather than defining definitive biological phenotypes, the present findings suggest the presence of three exploratory stress profiles that require validation in larger and independently recruited samples. In future research, the temporal stability of these profiles, their relationship with sporting outcomes and their responsiveness to interventions should be tested using repeated cortisol measurements, autonomic indicators, objective performance data and longitudinal designs [45].

## Figures and Tables

**Table 1:** Distribution of sporting, physiological and psychological characteristics of wheelchair basketball players.

Variables	Min.	Max.	X	S.d.
Weight	51.00	96.00	72.14	10.14
Height *	124.00	156.00	142.14	8.73
Age (years)	21.00	47.00	35.00	7.75
Sport Age (years)	4.00	17.00	9.79	3.81
Classification Point **	1.00	4.50	2.74	1.04
Cognitive Anxiety Pre	7.00	15.00	11.12	1.79
Bodily Anxiety Pre	5.00	12.00	8.79	1.51
Self-Confidence Pre	12.00	19.00	16.62	1.41
Perceived Stress Pre	2.00	7.00	3.65	1.61
Cortisol Pre (nmol/L)	6.00	15.00	10.03	2.10

* Height: Athlete’s height while seated in a wheelchair. ** functional classification system governed by the International Wheelchair Basketball Federation (IWBF).

**Table 2:** Distribution of sporting, physiological and psychological characteristics across the three stress reactor groups.

Variable	Low Reactor (*n* = 5)	High Reactor (*n* = 20)	Physiological Stress Reactor (*n* = 9)	Total (*N* = 34)
**Weight (kg)**	83.6 ± 13.35	70.8 ± 7.66	68.77 ± 9.84	72.14 ± 10.14
**Height (cm)**	139.40 ± 9.37	140.66 ± 11.67	143.5 ± 7.19	142.14 ± 8.73
**Age (years)**	43 ± 4.9	34.85 ± 6.61	30.89 ± 8.54	35 ± 7.75
**Sport Age (years)**	10.6 ± 2.61	9.95 ± 4.01	9 ± 4.15	9.79 ± 3.81
Classification Point *	1.9 ± 1.19	2.85 ± 1.04	2.94 ± 0.81	2.74 ± 1.04
**Cortisol Pre (nmol/L)**	7.4 ± 0.89	9.8 ± 1.61	12 ± 1.66	10.03 ± 2.10
**Cognitive Anxiety Pre**	9.8 ± 1.3	12.05 ± 1.43	9.78 ± 1.48	11.12 ± 1.79
**Bodily Anxiety Pre**	6.6 ± 1.14	9.65 ± 1.14	8.11 ± 0.60	8.79 ± 1.51
**Self-Confidence Pre**	17.8 ± 1.1	16.6 ± 1.14	16 ± 1.8	16.62 ± 1.41
**Perceived Stress Pre**	2.2 ± 0.45	3 ± 0.79	5.89 ± 1.05	3.65 ± 1.61
**Home Match, *n* (%)**	1 (20%)	9 (45%)	5 (55.6%)	17 (50%)
**Away Match, *n* (%)**	4 (80%)	11 (55%)	4 (44.4%)	17 (50%)
**Win, *n* (%)**	3 (60%)	10 (50%)	4 (44.4%)	17 (50%)
**Lose, *n* (%)**	2 (40%)	10 (50%)	5 (55.6%)	17 (50%)

* Functional classification system governed by the International Wheelchair Basketball Federation (IWBF).

**Table 3:** Analysis of Differences Among the Three Stress Reactor Groups.

Variable	Low Reactor (*n* = 5)	High Reactor (*n* = 20)	Physiological Stress Reactor (*n* = 9)	Kruskal–Wallis H(2)	ε^2^	95% CI ε^2^	Post hoc
**log(Cortisol)**	2.08 ± 0.13 ^a^	2.30 ± 0.13 ^b^	2.40 ± 0.17 ^c^	17.095 ***	0.518	[0.297, 0.720]	a < b < c
**Cognitive Anxiety**	10 ± 2 ^a^	12 ± 2 ^b^	10 ± 2 ^a^	13.900 **	0.421	[0.179, 0.655]	a < b; a = c; b > c
**Bodily Anxiety**	7 ± 1 ^a^	10 ± 1 ^c^	8 ± 0 ^b^	18.840 ***	0.571	[0.348, 0.771]	a < b, a < c,
**Self-Confidence**	18 ± 0 ^a^	17 ± 1.25 ^a^	16 ± 1 ^a^	5.266	0.160	[0.017, 0.450]	-
**Perceived Stress**	2 ± 0 ^a^	3 ± 0.25 ^a^	6 ± 2 ^b^	21.831 ***	0.662	[0.442, 0.812]	a < c, b < c,

*** *p* < 0.001. ** *p* < 0.01. Values are median ± IQR. The Kruskal–Wallis H test was used due to violations of the assumptions of normality. Epsilon-squared (0) = non-parametric effect size with bootstrap 95% confidence intervals (R = 1000). Post hoc comparisons were conducted using Dunn’s test with Bonferroni correction. Cliff’s delta effect sizes: all significant comparisons were classified as Large (|δ| > 0.474). Superscript letters indicate significant pairwise differences (*p* < 0.05); shared superscripts denote non-significant differences.

**Table 4:** Exploratory Logistic Regression Analysis of Classification Experience and Match Factors.

Variable	B	SE	OR	95% CI (Lower–Upper)	*p*
Intercept	−0.49	1.195	0.613	0.053–6.537	0.682
Classification High (≥3.5)	0.309	0.904	1.363	0.213–8.189	0.732
Sport Year	−0.126	0.116	0.882	0.690–1.099	0.277
Home Game (vs. Away)	1.327	0.862	3.771	0.746–23.800	0.124
Win (vs. Lose)	−0.287	0.825	0.751	0.140–3.835	0.728

Dependent: Physiological Stress Reactor (1) vs. Others (0), OR: Odds Ratio, CI: Confidence Interval, Given the small number of Physiological Stress Reactor cases (n = 9), the estimates should be interpreted cautiously.

## Data Availability

The data presented in this study are available upon request from the corresponding author. The data are not publicly available for further publication because of restrictions related to ongoing work.

## Abbreviations

The following abbreviations are used in this manuscript:

ARI	Adjusted Rand Index
CSAI-2	Competitive State Anxiety Inventory-2
HPA	hypothalamic–pituitary–adrenal
NMI	Normalised Mutual Information
PCA	Principal Component Analysis
CSAI-2R	Revised Competitive State Anxiety Inventory-2
